# Novel protective effect of the *FOXO3* longevity genotype on mechanisms of cellular aging in Okinawans

**DOI:** 10.1038/s41514-024-00142-8

**Published:** 2024-03-08

**Authors:** Trevor H. Torigoe, D. Craig Willcox, Michio Shimabukuro, Moritake Higa, Mariana Gerschenson, Anastasia Andrukhiv, Makoto Suzuki, Brian J. Morris, Randi Chen, Greg S. Gojanovich, Richard C. Allsopp, Bradley J. Willcox

**Affiliations:** 1 Institute for Biogenesis Research, Department of Anatomy, Biochemistry, and Physiology, John A. Burns School of Medicine, University of Hawaii, Honolulu, HI USA; 2 Department of Geriatric Medicine, John A. Burns School of Medicine, University of Hawaii, Honolulu, HI USA; 3 Department of Human Welfare, Okinawa International University, Ginowan, Okinawa Japan; 4 Okinawa Research Center for Longevity Science, Urasoe, Okinawa Japan; 5 Department of Research, Kuakini Medical Center, Honolulu, HI USA; 6 Department of Diabetes, Endocrinology and Metabolism, Fukushima Medical University School of Medicine, Fukushima, Fukushima, Japan; 7 Diabetes and Life-Style Related Disease Center, Tomishiro Central Hospital, Tomishiro, Okinawa Japan; 8 Department of Cell and Molecular Biology, John A. Burns School of Medicine, University of Hawaii, Honolulu, HI USA; 9 School of Medical Sciences, University of Sydney, Sydney, NSW Australia

**Keywords:** Cell biology, Risk factors

## Abstract

The genetic association of *FOXO3* genotypes with human longevity is well established, although the mechanism is not fully understood. We now report on the relationship of the *FOXO3* longevity variant *rs2802292* with telomere length, telomerase activity, *FOXO3* expression, and inflammatory cytokine levels in men and women. In agreement with earlier work, the *FOXO3* longevity variant conferred protection against telomere shortening of peripheral blood mononuclear cells from adults aged 55 years and older. This was accompanied by higher levels of telomerase activity in mononuclear cells for carriers of the longevity-associated *FOXO3 G*-allele of SNP *rs2802292* (*P* = 0.015). *FOXO3* mRNA expression increased slightly with age in both young (*P* = 0.02) and old (*P* = 0.08) *G*-allele carriers. Older female *G*-allele carriers displayed a modest decline in levels of pro-inflammatory cytokine IL-6 with age (*P* = 0.07). In contrast, older male *G*-allele carriers displayed an age-dependent increase in levels of anti-inflammatory cytokine IL-10 with age (*P* = 0.04). Thus, *FOXO3* may act through several different pro-longevity mechanisms, which may differ by age and sex.

## Introduction

Human aging is a multi-faceted process associated with increased risk for chronic disease, disability, and economic hardship. The U.S. Census Bureau projects that the ratio of the population aged 65-plus years will increase markedly to 1 in 5 Americans by the year 2030^1^. This demographic shift will increase the prevalence of age-related diseases, thus demanding a re-allocation of resources in healthcare and accompanying social services^2,3^. While specific mechanisms involved in human aging have yet to be fully elucidated, studies of the forkhead/winged helix box O type 3 (FOXO3) gene (*FOXO3*) have consistently demonstrated an association with human longevity. This has been replicated in multiple studies across diverse populations over the past 13 years and *FOXO3* is now the second most replicated gene for having variants associated with human longevity^4^.

FOXO3 is one of four isoforms that comprise the FOXO family of transcription factors in mammals. It is related to a large group of evolutionarily conserved homologous transcription factors linked with longevity in many diverse species, including *Caenorhabditis elegans*, *Hydra*, *Drosophila melanogaster*, rodents and humans^5–11^. The four mammalian isoforms, FOXO1, -3, -4, and -6, have varying and somewhat overlapping expression patterns in different tissues. *FOXO3* is expressed in multiple tissues throughout the body, including in blood (hematopoietic cells), heart, brain, liver, muscle, spleen, testes, and ovaries^12–14^. Studies in model organisms have demonstrated that FOXO3 (also termed FoxO3 in rodents and, in *C elegans*, daf-16) is a key regulator in multiple longevity-associated pathways, including those involved with energy homeostasis, autophagy, stem cell maintenance, and stress-resistance^6,7,15–17^.

Our research group was the first to report an association between *FOXO3* variants and human longevity—initially in a population cohort of American men of mainland Japanese and Okinawan-Japanese ancestry residing in Hawaii^12^. Such a genetic association has been independently replicated multiple times in other Asian and in European-ancestry populations^13,14,18,19^. The single nucleotide polymorphism (SNP) *rs2802292* was found to have the strongest association with longevity, with carriers of its protective *G*-allele having a 1.9-fold (*P* = 0.0003) increased probability of living past 95 years of age when compared to homozygote carriers of the non-protective, common *T*-allele^12^. More recently, we observed that this longevity-associated variant of *FOXO3* conferred substantial protection of telomeres as a function of age^20^.

Telomeres are DNA-protein complexes capping the end of chromosomes and protect the internal genetic material of somatic cells^21^. In human somatic cells, telomeres shorten with every replicative cycle at a rate of between 30 and 150 base pairs (bp)/year depending on the tissue and can serve as a cellular mechanism to determine the number of divisions a cell can undergo before entering senescence or apoptosis^22–26^. Shorter telomere length has been associated with greater risk for age-related diseases^26–28^ and telomere length may be a robust mechanism to assess biological age^29^. In a previous study, we demonstrated a protective effect on telomeres during aging linked to the *FOXO3* genotype, specifically in carriers of the *FOXO3 G*-allele^20^.

Pro- and anti-inflammatory factors must be maintained to effectively manage the aging process and not prematurely accelerate cells towards senescence^30–35^. The immune system must consistently balance cellular mechanisms that regulate baseline levels of inflammation with those that respond to and regulate the body’s acute inflammatory defenses from pathogens and tissue injury^30^. Many diseases commonly associated with aging exhibit elevated chronic, low-grade inflammation, a process referred to as “*inflammaging*”^31^. Imbalance between pro- and anti-inflammatory mechanisms can lead to elevated chronic inflammation and push cells towards senescence^32–35^. The immune system remains in a state of active surveillance throughout an individual’s lifetime^32^. Homeostatic regulation between pro- and anti-inflammatory factors are seen to be in greater balance throughout the aging process in those with greater longevity, such as centenarians^30^. We have found that subjects who were carriers of the longevity-associated minor (*G*) allele of *rs2802292* had significantly lower blood levels of TNF-α than the *TT* genotype^36^. This finding led us to the current, more detailed exploration of the effect of the longevity-associated *FOXO3* variant on inflammatory cytokine levels.

The objective of the present study was to investigate the effect of *FOXO3 rs2802292* longevity-associated *G*-allele carriers and *TT* genotype on telomere length, telomerase activity, and inflammatory cytokine levels (pro-inflammatory IL-1β, IL-2, IL-6, and TNF-α, and anti-inflammatory IL-10) during aging. To achieve this, a cohort of adult (age range 19–104 years) Okinawan-Japanese men and women was recruited. The Okinawan-Japanese population is an ideal study group due to the high percentage of long-lived individuals, including centenarians, less genetic diversity (little population stratification artifact), and relatively homogenous environment^37^.

## Results

### The *FOXO3* longevity-associated allele protects telomeres during aging

*FOXO3* genotype, telomere length and telomerase activity were assessed in a total population of 325 Okinawan-Japanese men and women of age range 19–104 years. Table 1 summarizes the number of subjects and ages of the study population. There were no significant differences in age between subjects with the *TT* genotype and *G*-carriers in the total population (*P* = 0.50) or between the sexes (female *P* = 0.68, male *P* = 0.61).

**Table 1:** Okinawan subject population for telomere length and telomerase activity

	Population (n)			Age (years)		
	*TT*	*G*-carrier	Total	*TT*	*G*-carrier	*P*-value
Female	87	79	166	57.6 ± 22.3	59.2 ± 22.3	0.68
Male	93	66	159	56.5 ± 20.7	58.2 ± 20.1	0.61
Total	180	145	325	57.2 ± 20.9	58.8 ± 21.3	0.50

The average age for *FOXO3 TT* and *G*-allele carriers was not significantly different in the total population (*P* = 0.50) and in both sexes (Female *P* = 0.68 and Male *P* = 0.61; Student’s *t* test).

**Fig. 1:**
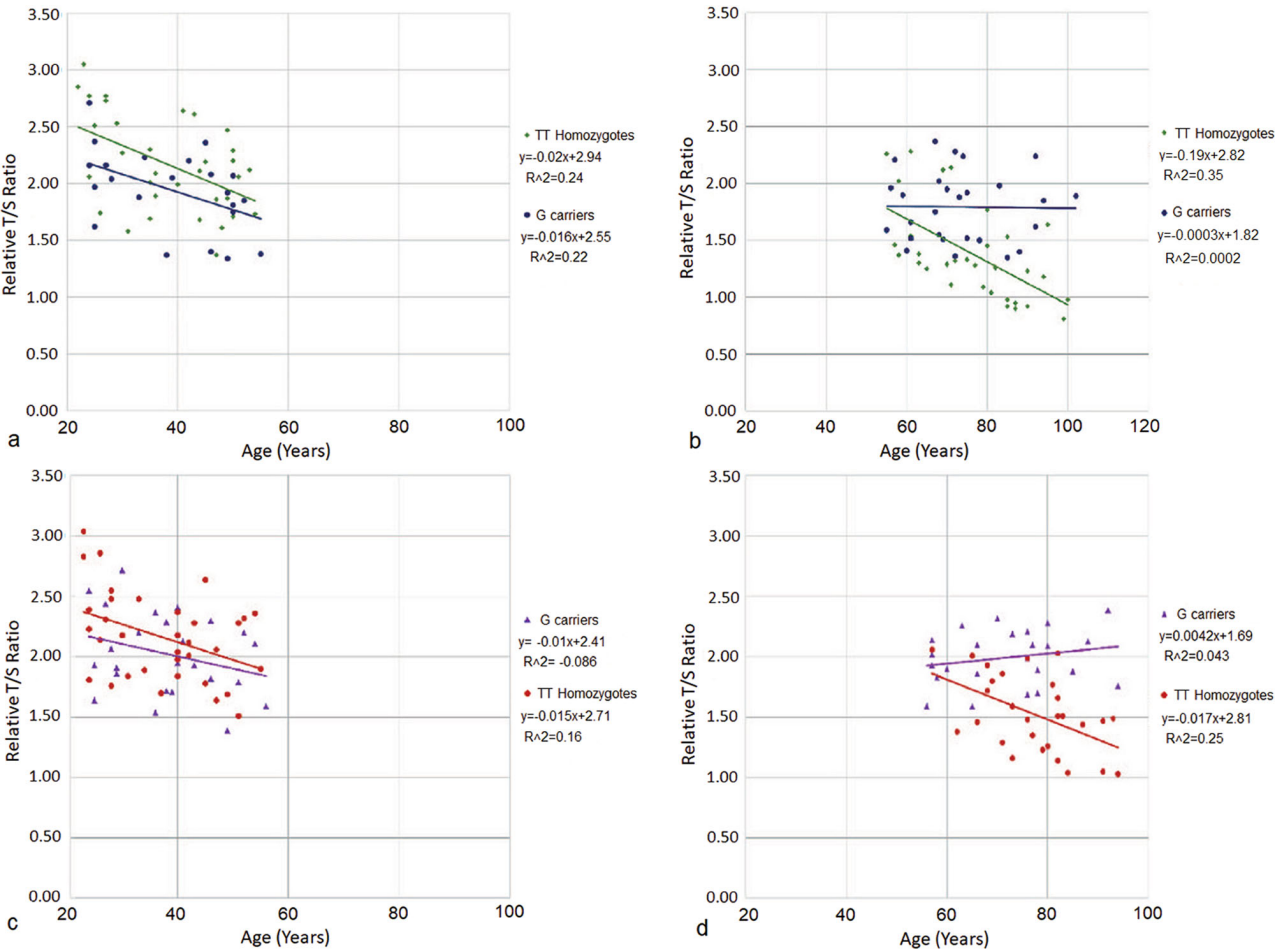
Effect of *FOXO3* Genotype on Telomere Length During Aging in Men and Women. The populations were divided into young (ages 19–54 years) and old (ages ≥55 years) for men (**a**, **b**) and women (**c**, **d**), respectively. For young participants, *FOXO3* genotype did not significantly affect telomere length as a function of age (*P* > 0.1 for men and women). In contrast, *FOXO3* genotype had a significant protective effect on telomere length in old male and female participants (*P* < 0.001). Telomere length was assessed using monochrome multiplex quantitative polymerase chain reaction (mmqPCR)^49^. Telomere length was determined as a telomere/single copy gene (T/S) ratio as a reference to C_t_ value of each respective gene expression reaction.

Leukocyte telomere length (LTL) was analyzed as a function of *FOXO3* genotype in both males and females (Fig. 1). In both sexes, carriers of the longevity-associated *G*-allele exhibited significant protection of telomeres cross-sectionally as compared to individuals having the *TT* genotype (*P* < 0.001 in both men and women).

To better assess the association of *FOXO3* genotype with telomere length and longevity, the total population was divided into younger adults (ages 19–54 years) and older adults (ages 55+ years). The young-old cutoff age of 55 years was determined using the average ages from the various study populations (Tables 1–3). Younger males and females both followed similar trends, with *TT* genotype subjects having, on average, longer telomeres at baseline than *G*-allele carriers (T/S ratio for males: *TT* = 2.54, *G*-allele carriers = 2.24; T/S ratio for females: *TT* = 2.43, *G*-allele carriers = 2.22). In the older adult group of males and females, telomeres were significantly protected cross-sectionally in individuals with the *G*-allele (T/S ratio/year for males: *G*-allele carriers = –0.0003, *TT* = –0.019; T/S ratio/year for females: *G*-allele carrier = 0.0042, *TT* = –0.017; *P* < 0.001, pooled sample) (Fig. 1).

**Table 2:** Okinawan subject population for *FOXO3* gene expression

	Population (n)			Age (years)		
	*TT*	*G*-carrier	Total	*TT*	*G*-carrier	*P* value
Female	67	52	119	58.0 ± 22.6	58.8 ± 22.1	0.86
Male	63	42	105	55.7 ± 23.0	59.2 ± 21.9	0.44
Total	130	94	224	57.7 ± 22.8	59.0 ± 22.0	0.67

The average ages between *TT* and *G*-allele carriers was not significantly different in the total population (*P* = 0.67) and in both sexes (Female *P* = 0.86 and Male *P* = 0.44; Student’s *t* test).

**Table 3:** Okinawan subject population for cytokine analysis

	Population (n)			Age (years)		
	*TT*	*G*-carrier	Total	*TT*	*G*-carrier	*P*-value
Female	72	69	141	60.6 ± 21.0	57.1 ± 21.4	0.34
Male	83	58	141	55.8 ± 20.7	59.2 ± 19.8	0.34
Total	155	127	282	58.0 ± 21.0	58.1 ± 20.7	0.99

The average age between *TT* and *G*-allele carriers was not significantly different in the total population and in both sexes (*P* > 0.1; Student’s *t* test).

### Carriers of the *FOXO3* longevity-associated allele retain higher levels of telomerase during aging

**Fig. 2:**
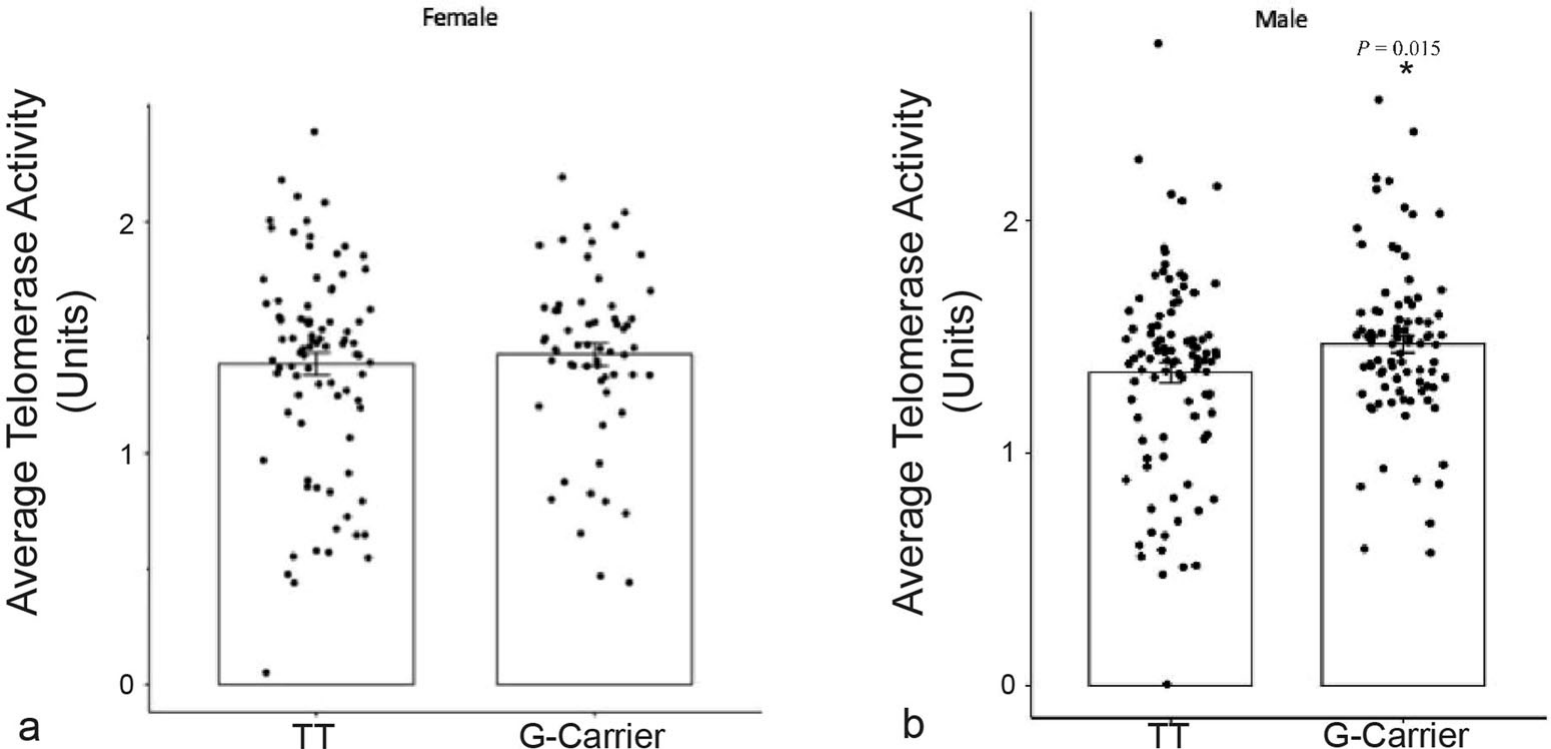
Effect of *FOXO3* Genotype on Mean Telomerase Activity for Young and Old Participants. Telomerase activity was measured in PBMC samples using the TRAP assay. Mean telomerase activity for carriers and non-carriers of the longevity-associated *FOXO3 G*-allele was compared for young (**a**; age 19–54 years) and old (**b**; age ≥55 years) participants. Significantly higher levels of telomerase activity were observed in *G*-allele carriers for old participants (*TT* = 94, *G*-allele carriers = 88; *P* = 0.015) relative to young (*TT* = 87, *G*-allele carriers = 59; *P* = 0.56*)*. (Student’s *t*-test). Error bars represent standard deviation. Three replicates were performed per sample.

To determine a possible correlation between telomerase activity as a mechanism of maintaining telomere lengths during aging, telomerase activity was analyzed in white blood cells as a function of *FOXO3* genotype. The same population studied in which telomeres were assessed was also used to measure telomerase activity. No difference in association of genotype with telomerase activity was seen between men and women (Supplementary Fig. 1, *P* > 0.05). Analysis of telomerase activity in young versus old adults revealed an age effect. Specifically, in the old population *G*-allele carriers exhibited higher telomerase activity (Fig. 2, *P* = 0.015), compared to individuals with the *TT* genotype. No difference in telomerase activity was observed between male and female *G*-allele carriers (Supplementary Fig. 1).

### Carriers of the *FOXO3* longevity-associated allele are better protected from age-associated decline in *FOXO3* expression than non-carriers

**Fig. 3:**
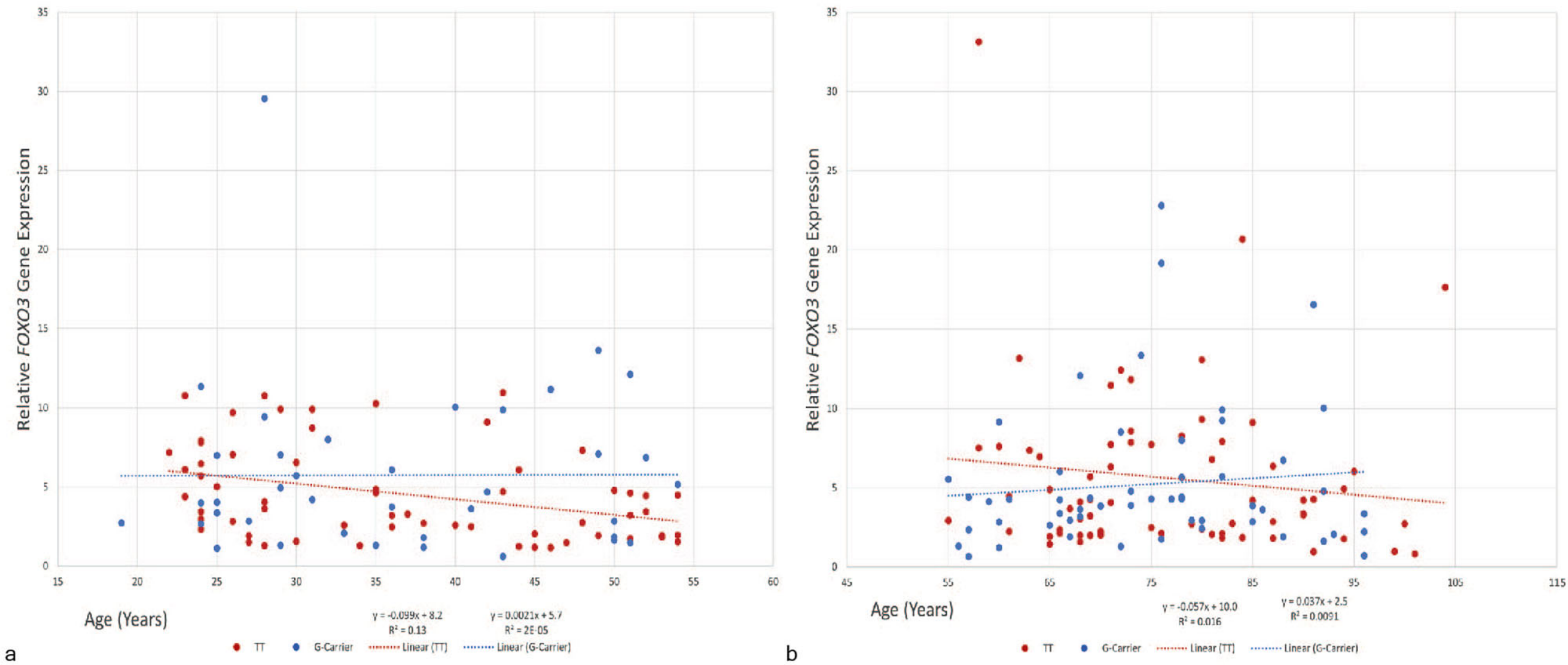
Effect of *FOXO3* Genotype on *FOXO3* Expression During Aging. *FOXO3* expression was analyzed for young (**a**, age 19–54 years) and old (**b**, age ≥ 55 years) participants using real time PCR. Possession of the longevity *FOXO3 G*-allele was associated with better retention of *FOXO3* gene expression as a function of age in young (**a**; *n* = 100, *TT* = 61, *G*-allele carriers = 39; *P* = 0.02), and with a trend toward significance in the old (**b**; *n* = 124, *TT* = 69, *G*-allele carriers = 55; *P* = 0.08) participants.

The *rs2802292* SNP is located within the non-coding intron 2 region of *FOXO3*^12^. In *C. elegans*, deletion of the *FOXO3* homolog *daf-16* results in extension of lifespan^6^. Table 2 summarizes the size and sex of the population of subjects used for the gene expression study. Despite limited power, the overall expression results demonstrated informative trends. For the study population, both young adults (ages 19–54 years; *n* = 100; *TT* = 61, *G*-allele carriers = 39) and older adults (ages 55+ years; *n* = 124, *TT* = 69, *G*-allele carrier = 55), carriage of the *G*-allele was associated with a borderline significant retention of *FOXO3* expression as a function of age (Fig. 3a, younger participants: *P* = 0.02; Fig. 3b: *P* = 0.08). For both men and women, the *FOXO3 G*-allele did not significantly affect the association between *FOXO3* expression and age when assessed over the entire age range (19–100+; Supplementary Fig. 2).

### Carriers of the *FOXO3* longevity-associated allele are protected from chronic inflammation, with different effects in men and women

In previous studies assessing the risk for coronary heart disease (CHD), protective *G*-genotypes were associated with lower CHD mortality in multiple populations, and lower inflammatory markers, in particular C-reactive protein^38^. Inflammatory cytokines were studied in a second subset of the total population, as summarized in Table 3.

**Fig. 4:**
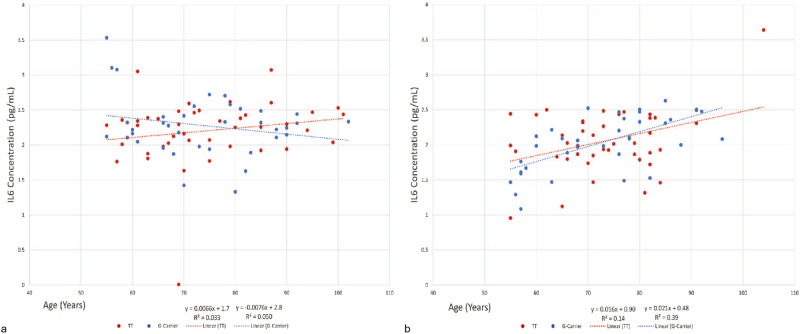
Effect of *FOXO3* Genotype on IL-6 Levels in Women and Men ≥ 55 years. Relative IL-6 levels were assessed as a function of genotype in the older female population (**a**) (*n* = 80, *TT* = 43, *G*-allele carriers = 37). Possession of the *G*-allele was associated with protection against increasing levels of the pro-inflammatory cytokine IL-6 as a function of age that approached significance when compared to individuals with the *TT* genotype (*P* = 0.07). Relative IL-6 levels were assessed as a function of genotype in the older male population (**b**) (*n* = 78, *TT* = 42, *G*-carriers = 36). Possession of the *G*-allele did not confer any benefit as a function of age when compared to the *TT* genotype (*P* > 0.05). A significant sex-specific effect was observed, namely, females demonstrated robust protection against increasing levels of IL-6 as a function of age as compared to males (*P* = 0.0006). No effect of *FOXO3* genotype on IL6 levels was observed in the young.

**Fig. 5:**
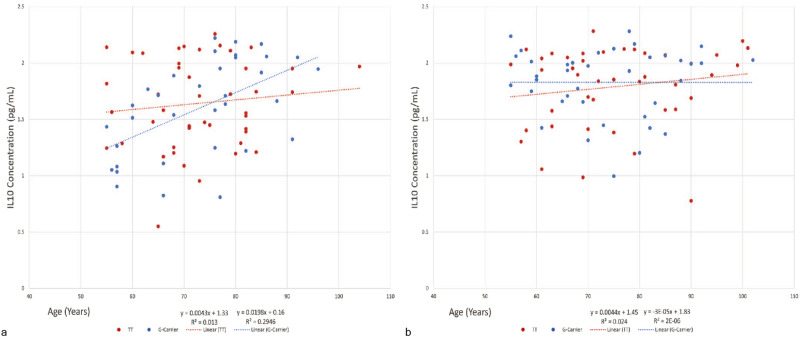
Effect of *FOXO3* Genotype on IL-10 Levels in Men and Women Aged ≥ 55 years. Relative IL-10 levels were assessed as a function of genotype in the old male population (**a**) (*n* = 78, *TT* = 44, *G*-carrier=34). Carriage of the protective *G*-allele correlated with enhanced increase in levels of the anti-inflammatory cytokine IL-10 with age compared to those lacking the *G*-allele (*TT* genotype) (*P* = 0.04). Relative IL-10 levels were assessed as a function of genotype in the older female population (**b**) (*n* = 80, *TT* = 42, *G*-carrier=38). Possession of the *G*-allele did not exhibit an association with increases of the anti-inflammatory cytokine when compared to that seen in individuals with the *TT* genotype (*P* > 0.05). A significant sex-specific effect was observed, namely, males demonstrated robust protection against decreasing levels of IL-10 as a function of age as compared to females (*P* = 0.007). No effect of *FOXO3* genotype on IL10 levels was observed in the young.

In subjects aged 55 years and older (*n* = 159), IL-6 and IL-10 plasma protein levels demonstrated trends as a function of age (Figs. 4 and 5). Specifically, for the pro-inflammatory cytokine IL-6 (Fig. 4), female carriers of the *G*-allele displayed a trend towards decreased cytokine levels when compared to females with the *TT* genotype (*P* = 0.07). While no such association was observed for older males, a highly significant sex-specific effect was observed when comparing female with male *G*-allele carriers (*P* = 0.0006). For the anti-inflammatory cytokine IL-10 in the older adults, male *G*-allele carriers showed a significantly greater increase in IL-10 plasma protein levels during aging (0.02 pg/mL/year), as compared to individuals with the *TT* genotype (0.0043 pg/mL/year; *P* = 0.04) or female *G*-allele carriers (–3.0 × 10^-5^ pg/mL/year; *P* = 0.007) (Fig. 5). No such association was observed for the older females (Fig. 5). There was no significant association of *FOXO3 G*-allele carrier frequency with cytokine levels as a function of age for IL-2, TNFα, and IL-1β (Supplementary Fig. 3), nor was there an association between the presence *FOXO3 G*-allele and age for men or women when assessed over the entire age range (19–100+; Supplemental Figs. 4 and 5).

## Discussion

The present study assessed the effect of the longevity-associated *G*-allele of *FOXO3* SNP *rs2802292* on telomeres, telomerase, *FOXO3* expression, and inflammatory cytokine levels in an Okinawan-Japanese cohort. In agreement with our previous study^20^, we observed a protective effect of having a *FOXO3 rs2802292 G*-allele on telomeres (Fig. 1). We also demonstrated for the first time that telomerase activity is greater in *FOXO3 G*-allele carriers than in those with the *TT* genotype, particularly in the older adult population (aged ≥55 years) (Fig. 2). Furthermore, expression of *FOXO3* mRNA was found to increase during aging for *FOXO3 G*-allele carriers, but not for the *TT* genotype. Finally, levels of two inflammatory cytokines were found to differ by *FOXO3* genotype in a sex-specific manner. Elderly female *FOXO3 G*-allele carriers were protected against age-related increase in levels of the pro-inflammatory cytokine IL-6, whereas older adult male *FOXO3 G*-allele carriers displayed gradual age-related increase in the levels of the anti-inflammatory cytokine IL-10, as compared to the *TT* genotype.

The results of this study expand upon our previous findings showing the protective effects of the *FOXO3 G* (longevity-associated) allele on telomeres in a cross-sectional analysis^20^. Importantly, we were able to assess the association of the *FOXO3 G*-allele separately for both men and women. As shown in Fig. 1, the *FOXO3 G*-allele was associated with protection of telomeres in the older adult population for both men and women equally. Interestingly, telomere length in older adult women was generally longer than in older men, for both *FOXO3 G*-allele carriers (female: 2.03 kb, male: 1.80 kb) and those with the *TT* genotype (female: 1.48 kb, male: 1.31 kb). This is consistent with previous studies comparing telomere length for men and women^39^.

Although telomerase levels did not increase with age in the total sample population (Supplemntary Fig. 1), average telomerase activity was found to be significantly higher in older adult *FOXO3 G*-allele carriers compared with individuals having the *TT* genotype (*P* = 0.015). In the young population (ages 19–54 years), this relationship was not statistically significant (*P* = 0.56). These findings may help elucidate a mechanism by which the longevity-associated *FOXO3* genotype may protect telomeres during human aging^40,41^. Notably, ablating telomerase activity in rodent models leads to more rapid decline in telomere length with age^40^. In contrast, transgenic enhancement of telomerase in hematopoietic stem cells has the opposite effect^42^. Thus, increased levels of telomerase activity in *G*-allele carriers likely explains, at least in part, the associated protection of telomeres. Whether FOXO3 directly or indirectly regulates the expression of Tert, the catalytic component of telomerase, and whether the *FOXO3 G*-allele is associated with enhanced levels of telomerase in hematopoietic stem cells will need to be ascertained in future studies.

Previously, *FOXO3* expression has been studied in different tissues^9–11^, but never before in relation to telomerase dynamics as a mechanism of longevity. Here we have observed, for the first time, a modest but significant increase in *FOXO3* expression with age in *FOXO3 G*-allele carriers, whereas *FOXO3* expression decreased with age in individuals with the *TT* genotype (Fig. 3). These results are in agreement with our previous observations of elevated *FOXO3* expression in H_2_O_2_ stressed and serum deprived lymphoblastoid cell lines established from *FOXO3 G*-allele carriers as compared to cell lines established from non-carriers^43^.

In the current study, we found significant sex-specific associations of the *FOXO3 G*-allele with levels of inflammatory cytokines IL-6 and IL-10 as a function of age in older patients (Figs. 4 and 5). Plasma levels of the pro-inflammatory cytokine IL-6 decreased during aging in the older (ages 55+ years) female *G*-allele carriers (Fig. 4, *P* = 0.07), in contrast to a gradual increase in IL-6 levels with age in males (Fig. 4, *P* = 0.0006). IL-6 levels normally increase with age^44,45^, as was seen in our older adult male and female *TT*-genotype populations. The decreasing IL-6 levels in the older adult female *G*-allele carriers with age suggests a moderating effect of *G*-allele carriage on expression of this pro-inflammatory cytokine. Interestingly, we observed the opposite effect of the *FOXO3 G*-allele on the anti-inflammatory cytokine IL-10 in men. Previous studies have demonstrated negative to static changes in IL-10 levels with increase in age^45,46^. In contrast, for older adult males, carriage of the *FOXO3 G*-allele was associated with an increase in IL-10 levels with age (Fig. 5, *P* = 0.04), whereas IL-10 levels remained relatively static during aging in the older females (*P* > 0.1). Direct comparison of the relationship between age and IL-10 levels between female and male *G*-allele carriers suggested a significant sex-specific effect (*P* = 0.007).

In earlier work from the Kuakini Honolulu Heart Program (HHP), the levels of two inflammatory markers, C-reactive protein (CRP) and TNF-α, were assessed in both *FOXO3 G*-allele carriers and non-carriers^36,47^. In that preliminary study, we observed lower levels of the pro-inflammatory cytokine TNF-α in older adult *G*-allele carriers aged 70–90 years (*P* = 0.008). The results from the present study demonstrate a protective (anti-inflammatory) effect of the *FOXO3* longevity genotype, although for different cytokines in men than in women. The theory of inflammaging is that basal inflammatory levels increase with aging, and that subjects better able to manage the pro- vs anti-inflammatory mediators are able to increase cellular lifespan^34^. In light of this theory, we also assessed the ratio of TNF-α and IL-10 levels in both males and females aged 55 years and older. In males, initial assessment showed lower ratios of TNF-α:IL-10 in carriers of the *FOXO3* longevity-associated *G*-allele compared to those with the *TT* genotype. The same trend was not demonstrated in women, suggesting that the benefit of carriage of the *rs2802292 G*-allele is specific to males.

We propose a model wherein the *FOXO3 G*-allele has an indirect protective effect on telomeres during aging. Chronic inflammation begins in middle-aged individuals and is known to drive hematopoietic cell turnover. We have shown in prior studies^48^ and here (Figs. 4 and 5) that older (>50 years) carriers of the *FOXO3* longevity associated *G*-allele have a reduced inflammatory cytokine profile. This in turn could provide protection of telomeres during aging in the elderly by reducing telomere shortening associated with cell division. Further, we observed a modest but significant increase in telomerase activity in hematopoietic cells from middle aged and older individuals (Fig. 2). While the mechanism accounting for this enhanced telomerase activity is unknown, it too could contribute to the reduced telomere shortening with age in peripheral blood cells from the elderly. In younger donors, hematopoietic cell turnover is likely substantially more frequent than in older individuals, due to growth, higher metabolic rate and other factors, thereby accounting for the more accelerated decrease in telomere length per year in these individuals regardless of *FOXO3* genotype.

While the *FOXO3* longevity-associated SNP *rs2802292* site does not match any known transcription factor binding site, we have hypothesized previously that it may act in a haplotype with other SNPs in *FOXO3* intron 2 that are in linkage disequilibrium with *rs2802292* and which are located within known transcription factor binding sites^48^. Furthermore, we have shown that when human cells are stressed in vitro, *FOXO3* forms a tight cluster with other neighboring genes^48^. We hypothesize that this drives *FOXO3* expression via a mechanism involving the *FOXO3* haplotype (i.e., by a super-enhancer or interactome effect)^48^. We further hypothesize that cells from *FOXO3 G*-allele carriers have enhanced levels of *FOXO3* activation (expression) during stress relative to cells from *TT* carriers^43,48^.

In conclusion, our findings suggest that the mechanism of the protective effect of the *FOXO3* longevity-associated genotype against mortality may differ slightly between men and women. The gender specific difference in the effect of the *FOXO3 G*-allele on inflammatory cytokine levels warrants further investigation in other populations.

## Methods

### Study design and clinical cohorts

Male and female participants (*n* = 320) ranging in age from 19 to 104 years were recruited between May 2018 and July 2019 during annual health examinations through Tomishiro Central Hospital (Tomishiro City, Okinawa, Japan) and affiliated clinics and facilities throughout the Okinawa prefecture. Subjects were recruited during nationally required annual health screening examinations, thereby mitigating potential bias towards healthier participants. Written informed consent was obtained from each subject. Principal inclusion criteria focused on healthy individuals over the age of 18 years. Subjects were excluded from participation if they were (a) aged <18 years, (b) had a recent medical complication, (c) exhibited severe dementia or an inability to comprehend the informed consent, (d) had a known genetic disease or disability, or (e) were restricted from participation by the subject’s attending physician. The study was conducted following approval by the Ethics Committees from Tomishiro Central Hospital (H25R008), Fukushima Medical University (#30167) and followed all relevant ethical regulations including the Declaration of Helsinki.

### Sample collection

12.5 milliliters (mL) of peripheral blood were collected, in addition to the usual amount of blood collected, during the annual health screening examination. This was apportioned as follows: 10 mL in EDTA vacuum tubes and 2.5 mL in PAXgene Blood RNA tubes (BD Biosciences). Following collection, EDTA tubes were stored at 4 °C for up to 16 days before being shipped at 4 °C to the John A. Burns School of Medicine (JABSOM) at the University of Hawaii for processing. PAXgene tubes, used for isolation of RNA and *FOXO3* mRNA expression analysis, were frozen at –80 °C for batch shipment to JABSOM on dry ice and later thawed on ice to minimize damage to the white blood cells. All samples were shipped on or before September 2019.

Two mL of Dulbecco’s phosphate buffered saline (DPBS) with calcium and magnesium (ThermoFisher Scientific) were added to 2 mL of whole blood and mixed at room temperature. Three mL of Ficoll-Paque PREMIUM (GE Healthcare/Cytiva) were added to the bottom of a new 10 mL centrifuge tube. Four mL of the diluted blood sample was carefully layered on top of the Ficoll-Paque Premium, ensuring that there was no mixing between the layers, before being centrifuged at 400 × *g* for 40 min with the brake turned off. After centrifugation, the upper layer containing the plasma was removed. Next, the mononuclear cell layer was removed from the centrifuge tube and added to a new tube. Cells of the mononuclear fraction were counted and used for protocols of (1) *FOXO3A rs2802292* genotyping and telomere length analysis (3.0 × 10^6^ cells) or (2) telomere repeat amplification protocol (TRAP) (5.0 × 10^5^ cells). Cells designated for the TRAP protocol were further washed in PBS and lysed with 3-[(3-cholamidopropyl) dimethylammonio]-1-propanesulfonate (CHAPS) lysis buffer (ThermoFisher Scientific).

### Genotyping

Subjects were genotyped for *FOXO3 rs2802292* SNP genotype using an amplification-refractory mutation system, allele-specific, polymerase chain reaction (PCR). PCR was performed on genomic DNA (125 ng) with the following primers: forward outer (“rs2802292_FO”), 5’- GAAACTGAGGCTAACAGCTGGGTCTGGCCC-3’, reverse outer (“rs2802292_RO”), 5’-AGCTGATGCTCCTCAACGAAACCACCTTAC-3’, reverse *G*-specific (“rs2802292_RG”), 5’-GGACCCCTTCATCTGTCACACAGAGGCTCC-3’, and forward *T*-specific (“rs2802292_FT”), 5’-CTGTTGCTCACAAGAGCTCAGGGCTGGGCT-3’. Final concentrations of the outer primers and allele-specific primers were 500 nM and 1 μM, respectively. PCR involved 30 cycles and PCR products were resolved on 3% agarose gels with 1x sodium borate buffer.

### Measurement of telomere length and telomerase activity

Telomere length was assessed using monochrome multiplex quantitative PCR (mmqPCR)^49^. Twenty (20) nanograms (ng) of sample genomic DNA were used in each reaction with SsoAdvanced Universal SYBR Green Supermix and specifically designed primers that anneal to telomere repeat sequences for amplification (Telg, 5’-ACACTAAGGTTTGGGTTTGGGTTTGGGTTTGGGTTAGTGT-3’, and Telc, 5-TGTTAGGTATCCCTATCCCTATCCCTATCCCTATCCCTAACA). Human beta-globin (HBG) (Hbgu, 5-CGGCGGCGGCGGCGCGGGCTGGCGGCTTCATCCACGTTCACCTTG-3’, Hbgd 5’-GCCCGGCCCGCCGCGCCCGTCCCGCCGGAGGAGAAGTCTGCCGTT-3’) was used as the reference single-copy gene to standardize the relative expression of telomere results. Thermal cycling was run for 30-cycles 1 cycle at 95 °C for 15 min, 2 cycles of 94 °C for 15 s and 49 °C for 15 s, and 32 cycles of 94 °C for 15 s, 62 °C for 10 s 74 °C for 15 s with signal acquisition of the telomere template, 84 °C for 10 s, 88 °C for 15 s with signal acquisition of the single-copy gene. Telomere length was determined as telomere/single copy gene (T/S) ratio as a reference to *C*_t_ value of each respective PCR. All analyses were performed in duplicate on a Bio-Rad real-time PCR machine.

Telomerase activity was assessed using the TRAPeze kit telomere repeat amplification protocol (TRAP) (EMD Millipore) following the manufacturer’s guidelines. 5 × 10^5^ cells were isolated and suspended in 200 μL of 1x CHAPS lysis buffer, incubated on ice for 30 min before centrifugation at 12,000 × *g* for 20 min at 4 °C. Supernatant was removed and transferred to a new 1.5 mL tube and stored at −80 °C until analysis. TRAP assay was performed using radioisotopic detection with γ-32-ATP (3000 Ci/mmol, 10 mCi/mL). End-labeling of the TS primer utilized components of the TRAPeze kit. The mixture was incubated for 20 min at 37 °C, then five min at 85 °C. The mastermix for PCR amplification was created using the components from the TRAPeze kit. Each sample was incubated at 30 °C for 30 min before PCR amplification. Two μL of cell extract was used in each reaction. After PCR, 25 μL of each sample was loaded on a 10% non-denaturing PAGE gel in 0.5X TBE buffer. The gel was dried and exposed to a phosphor image screen (GE) before being visualized and quantified using ImageQuant software version 5.1 (GE) and a Typhoon Variable Imager (Amersham Biosciences).

### *FOXO3* gene expression

Messenger RNA was isolated from mononuclear cell samples according to the manufacturer’s specifications. One microgram (µg) of mRNA was converted to cDNA using the iScript gDNA Clear cDNA Synthesis kit (BioRad). Quantitative PCR was performed using SsoAdvanced Universal SYBR Green Supermix (BioRad). One-hundred nanograms (ng) of cDNA and *FOXO3* primers designed to span intron 2 were used for PCR (forward primer, 5’-AACGTGGGGAACTTCACTGG-3’, and reverse primer, 5’-TTTGAGGGTCTGCTTTGCCC-3’). The hypoxanthine-guanine phosphoribosyltransferase gene (*HPRT*) was used as reference gene (forward primer, 5’-CAGGGATTTGAATCATGTTTGTGTC-3’; reverse primer, 5’-ACTGGCGATGTCAATAGGACTC-3’). PCR involved 40 cycles and was followed by a melt curve analysis. *FOXO3* expression was measured as a fold-change relative to *HPRT*. Cycle threshold difference (ΔC_t_) was determined by subtracting the cycle threshold of *HPRT* from the cycle threshold of *FOXO3* (Equation 1). Fold change was found by applying the base 2 to the power of minus ΔC_t_ (Equation 2).

1 $$\Delta {Ct}={{Ct}}_{{FOXO}3}-{{Ct}}_{{HPRT}}$$

2 $${Fold}\,{change}={2}^{-\Delta {Ct}}$$

### Inflammatory cytokine analysis

Plasma samples were collected in EDTA tubes from the same specimens obtained for genotyping, telomere length, telomerase activity analysis. The plasma was separated before the FICOL separation step by centrifugation and stored in 1 mL aliquots at −80 °C before analysis. Levels of cytokines IL-1β, IL-2, IL-6, IL-10, and TNF-α were measured in each plasma sample in duplicate using a Milliplex MAP Human High Sensitivity T Cell Panel (MilliporeSigma, Burlington, MA) on a Luminex 200 System (Luminex Corp, Austin, Texas)^50^. 25 μL of each sample was run in duplicate on a 96-well plate with a set of standards and internal control samples. 25 μL of antibody beads were used with each reaction and mixed into each sample, standard, and internal control. The plate was wrapped in foil and incubated overnight (at least 18 h) at 4 °C. After the incubation, the plate was placed on a magnet and washed before adding 50 μL of Detection Antibodies (DA) to each well. The plate was then placed on a shaker and incubated at room temperature for 60 min. After incubation, 50 μL of SAV-PE were added to each well. The plate was sealed, covered for protection from light and left on a shaker to incubate for 30 min, after which, another wash step was performed. 150 μL of sheath fluid was added to each well and the plate was protected from light and incubated at room temperature for five min before reading by the Luminex 200 system.

### Statistical analysis

Age-related effects on telomere length, telomerase activity, *FOXO3* expression, and cytokine activity were assessed using least squares linear regression (generalized linear model) for each *FOXO3 rs2802292* genotype and sex (SAS version 9.2; SAS Institute, Inc., Cary, North Carolina). Mean values of telomerase activity were compared between groups by *FOXO3 rs2802292* genotype, sex or age demographic (young: 19–54 years, or old: 55–104 years) using Student’s *t* test.

### Reporting summary

Further information on research design is available in the Nature Research Reporting Summary linked to this article.

### Supplementary information

Supplementary Figure File

Reporting Summary

## Data Availability

All data collected and used for the analysis are available via Excel file in Supplemental Materials. The corresponding author may be contacted to request the raw data and/or materials generated during this study.

## References

[1] Colby, S. L. & Ortman, J. M. *Projections of the Size and Composition of the U.S. Population:* 2014 to 2060. https://www.census.gov/library/publications/2015/demo/p25-1143.html (3AD).

[2] Canadian Health Services Foundation. (2003). The aging population will overwhelm the health care system. J. Health Serv. Res. Policy.

[3] Segal-Gidan F (2002). Who will care for the aging American population?. JAAPA.

[4] Butler RN (2004). The aging factor in health and disease: the promise of basic research on aging. Aging Clin. Exp. Res..

[5] Greer EL, Brunet A (2005). FOXO transcription factors at the interface between longevity and tumor suppression. Oncogene.

[6] Kenyon C, Chang J, Gensch E, Rudner A, Tabtiang R (1993). A *C. elegans* mutant that lives twice as long as wild type. Nature.

[7] Nebel A, Bosch TCG (2012). Evolution of human longevity: lessons from Hydra. Aging.

[8] Bosch TCG, Anton‐Erxleben F, Hemmrich G, Khalturin K (2010). The hydra polyp: nothing but an active stem cell community. Dev. Growth Differ..

[9] Giannakou ME, Partridge L (2004). The interaction between FOXO and SIRT1: tipping the balance towards survival. Trends Cell Biol..

[10] Webb AE, Kundaje A, Brunet A (2016). Characterization of the direct targets of FOXO transcription factors throughout evolution. Aging Cell.

[11] Martins R, Lithgow GJ, Link W (2015). Long live FOXO: unraveling the role of FOXO proteins in aging and longevity. Aging Cell.

[12] Willcox BJ (2008). FOXO3A genotype is strongly associated with human longevity. Proc. Natl. Acad. Sci. USA.

[13] Anselmi CV (2009). Association of the FOXO3A locus with extreme longevity in a Southern Italian centenarian study. Rejuv. Res..

[14] Flachsbart F (2009). Association of FOXO3A variation with human longevity confirmed in German centenarians. Proc. Natl. Acad. Sci. USA.

[15] Warr MR (2013). FOXO3A directs a protective autophagy program in haematopoietic stem cells. Nature.

[16] Miyamoto K (2007). Foxo3a is essential for maintenance of the hematopoietic stem cell pool. Cell Stem Cell.

[17] Miyamoto K (2008). FoxO3a regulates hematopoietic homeostasis through a negative feedback pathway in conditions of stress or aging. Blood.

[18] Li Y (2009). Genetic association of FOXO1A and FOXO3A with longevity trait in Han Chinese populations. Hum. Mol. Genet..

[19] Soerensen M (2010). Replication of an association of variation in the FOXO3A gene with human longevity using both case–control and longitudinal data. Aging Cell.

[20] Davy PMC (2018). Minimal shortening of leukocyte telomere length across age groups in a cross-sectional study for carriers of a longevity-associated *FOXO3* allele. J. Gerontol. A Biol. Sci. Med. Sci..

[21] Olovnikov AM (1973). A theory of marginotomy. The incomplete copying of template margin in enzymic synthesis of polynucleotides and biological significance of the phenomenon. J. Theor Biol..

[22] Cawthon RM, Smith KR, O’Brien E, Sivatchenko A, Kerber RA (2003). Association between telomere length in blood and mortality in people aged 60 years or older. Lancet.

[23] Willeit P (2010). Cellular aging reflected by leukocyte telomere length predicts advanced atherosclerosis and cardiovascular disease risk. Arterioscler. Thromb. Vasc. Biol..

[24] Takubo K (2000). Telomere shortening with aging in human liver. J. Gerontol. A Biol. Med. Sci..

[25] Fitzpatrick AL (2007). Leukocyte Telomere length and cardiovascular disease in the cardiovascular health study. Am. J. Epidemiol..

[26] Brouilette SW (2007). Telomere length, risk of coronary heart disease, and statin treatment in the West of Scotland primary prevention study: a nested case-control study. Lancet.

[27] Zee RYL, Castonguay AJ, Barton NS, Germer S, Martin M (2010). Mean leukocyte telomere length shortening and type 2 diabetes mellitus: a case-control study. Transl. Res..

[28] Brouilette S, Singh RK, Thompson JR, Goodall AH, Samani NJ (2003). White cell telomere length and risk of premature myocardial infarction. Arterioscler. Thromb. Vasc. Biol..

[29] Bodnar AG (1998). Extension of lifespan by introduction of telomerase into normal human cells. Science.

[30] Monti D, Ostan R, Borelli V, Castellani G, Franceschi C (2017). Inflammaging and human longevity in the omics era. Mech. Ageing Dev..

[31] Franceschi C (2000). Inflamm‐aging: an evolutionary perspective on immunosenescence. Ann. NY Acad. Sci..

[32] Pinti M (2014). Circulating mitochondrial DNA increases with age and is a familiar trait: implications for “inflamm‐aging”. Eur. J. Immunol..

[33] Oishi Y, Manabe I (2016). Macrophages in age-related chronic inflammatory diseases. NPJ Aging Mech. Dis..

[34] Franceschi C, Garagnani P, Vitale G, Capri M, Salvioli S (2017). Inflammaging and ‘garb-aging’. Trends Endocrinol. Metab..

[35] Minciullo PL (2016). Inflammaging and anti-inflammaging: the role of cytokines in extreme longevity. Arch. Immunol. Ther. Exp..

[36] Willcox BJ (2017). Longevity-Associated *FOXO3* genotype and its impact on coronary artery disease mortality in Japanese, Whites, and Blacks: A prospective study of three American populations. J. Gerontol. A Biol. Sci. Med. Sci..

[37] Bendjilali N (2014). Who are the Okinawans? Ancestry, genome diversity, and implications for the genetic study of human longevity from a geographically isolated population. J. Gerontol. A Biol. Sci. Med. Sci..

[38] Willcox BJ (2016). The FoxO3 gene and cause‐specific mortality. Aging Cell.

[39] Gardner M (2014). Gender and telomere length: systematic review and meta-analysis. Exp. Gerontol..

[40] Lee HW (1998). Essential role of mouse telomerase in highly proliferative organs. Nature.

[41] Broccoli D, Young JW, Lange TD (1995). Telomerase activity in normal and malignant hematopoietic cells. Proc. Natl. Acad. Sci. USA.

[42] Allsopp RC, Morin GB, DePinho R, Harley CB, Weissman IL (2003). Telomerase is required to slow telomere shortening and extend replicative lifespan of HSCs during serial transplantation. Blood.

[43] Donlon TA, Willcox BJ, Morris BJ (2017). *FOXO3* cell resilience gene neighborhood. Aging.

[44] Milan-Mattos JC (2019). Effects of natural aging and gender on pro-inflammatory markers. Braz. J. Med. Biol. Res..

[45] Koelman L, Pivovarova-Ramich O, Pfeiffer AFH, Grune T, Aleksandrova K (2019). Cytokines for evaluation of chronic inflammatory status in ageing research: reliability and phenotypic characterization. Immun. Ageing.

[46] Forsey RJ (2003). Plasma cytokine profiles in elderly humans. Mech. Ageing Dev..

[47] Morris BJ, Willcox DC, Donlon TA, Willcox BJ (2015). FOXO3: a major gene for human longevity - A mini-review. Gerontology.

[48] Donlon TA (2017). FOXO3 longevity interactome on chromosome 6. Aging Cell.

[49] Cawthon RM (2009). Telomere length measurement by a novel monochrome multiplex quantitative PCR method. Nucleic Acids Res..

[50] Shikuma CM (2014). The role of HIV and monocytes/macrophages in adipose tissue biology. J. Acquir. Immune. Defic. Syndr..

