# Supplementary material for: Novel protective effect of the FOXO3 longevity genotype on mechanisms of cellular aging in Okinawans
Source: NPJ Aging. 2024 Mar 8;10(1):18. doi: 10.1038/s41514-024-00142-8 (PMC10923797; doi:10.1038/s41514-024-00142-8)
Supplement: Supplementary file 1 — Supplementary Figure File [file 41514_2024_142_MOESM1_ESM.pdf]

Novel Protective Effect of the FOXO3 Longevity Genotype on Mechanisms of Cellular Aging in Okinawans – Supplementary Figure File

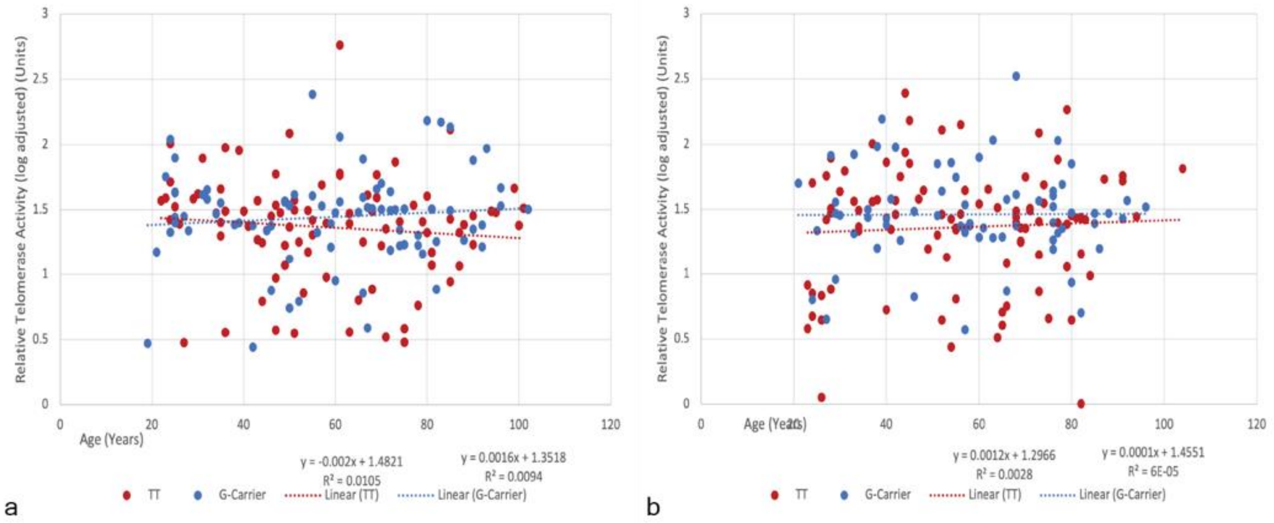

Supplementary Figure 1: Effect of *FOXO3* Genotype on Telomerase Activity During Aging
Telomerase activity was assessed as a function of age for *FOXO3 rs2802292* alleles (**a**; n=143, *TT*=75, *G*-carriers=68) in **women** (aged (19–102 years). *FOXO3* genotype was not associated with level of telomerase activity as a function of age (*P*=0.69). The results for **men** (aged 21–104 years) is shown in panel **b** (n=144, *TT*=85, *G*-carrier = 59). Similar to women, *FOXO3* genotype was not associated with level of telomerase activity as a function of age in men (*P*=0.59).

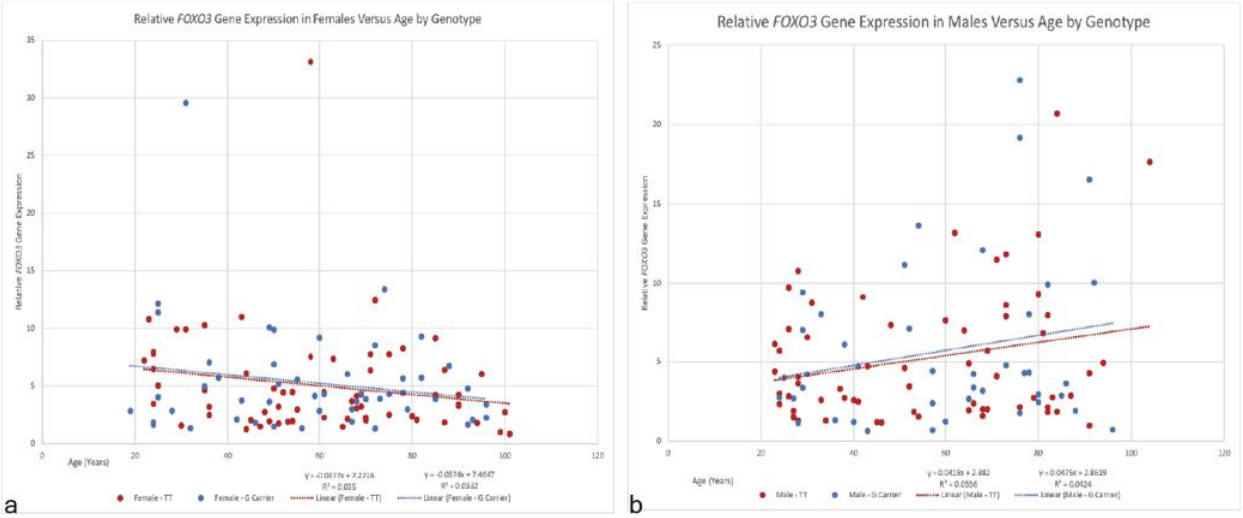

Supplementary Figure 2: Effect of *FOXO3* Genotype on *FOXO3* Gene Expression in Men and Women
FOXO3 mRNA expression versus age for the *FOXO3 rs2802292* alleles in women (All n=103, *TT*=63, *G*-carrier = 42). Carriers of the longevity-associated *FOXO3 G*-allele did not exhibit an age-related increase in gene expression (women: *P*=0.21; men: *P*=0.92).

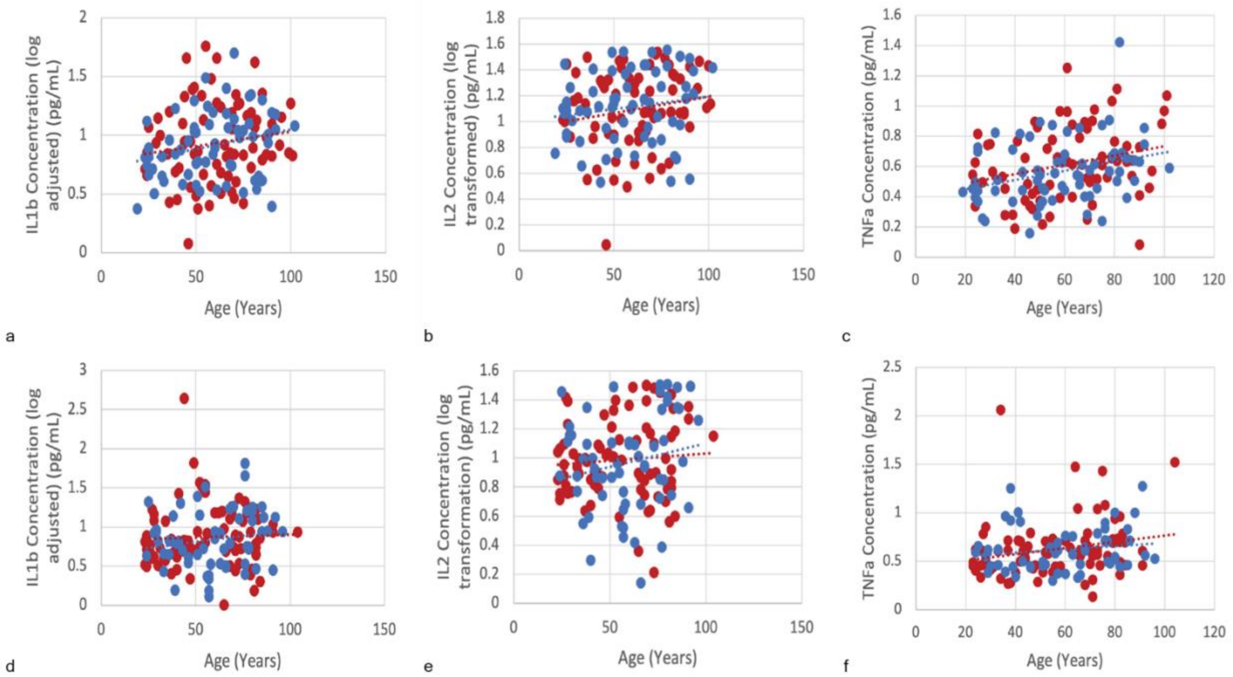

Supplementary Figure 3: Effect of *FOXO3* Genotype on Levels of IL=1β, IL-2, and TNFα Versus Age in Men and Women
IL-1β, IL2 and TNFα cytokine levels were analyzed as function of age for each *FOXO3* genotype in women (**a**) IL-1β (*P*=0.35), (**b**) IL-2 (*P*=0.54), (**c**) TNFα (*P*=0.98), and in men (**d**) IL-1β (*P*=0.48), (**e**) IL-2 (*P*=0.40), (**f**) TNFα (*P*=0.51). In each part of the figure, red dot-points represent carriers of the *TT* genotype and blue dot-points represent *G*-allele carriers. Red lines represent regression lines for carriers of the *TT* genotype while blue lines represent regression lines for *G*-allele carriers.

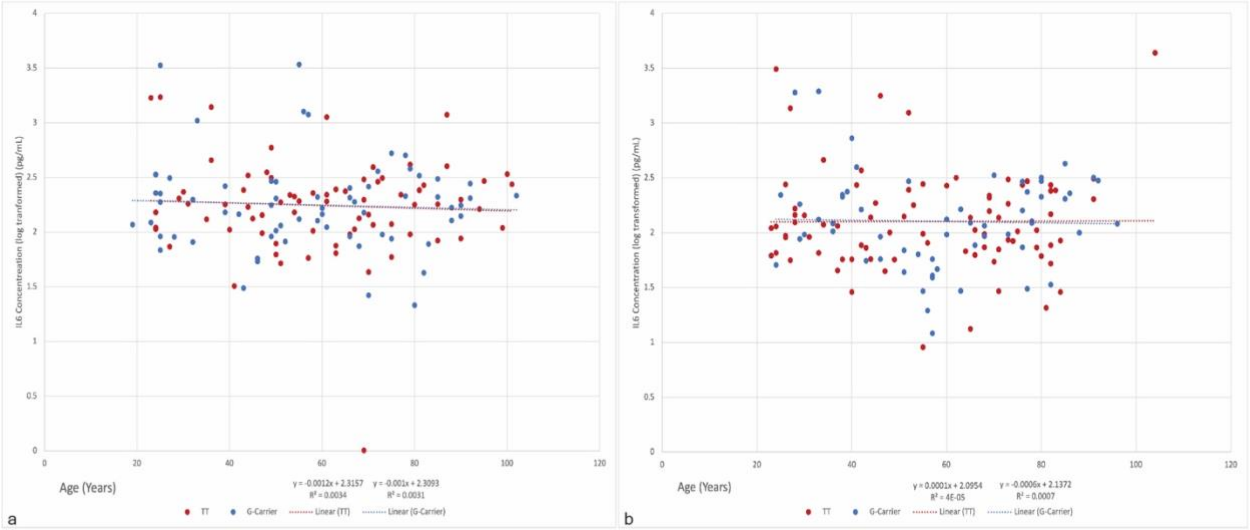

Supplementary Figure 4: Effect of *FOXO3* Genotype on Levels of IL6
IL6 cytokine levels were analyzed as function of age for each *FOXO3* genotype in women (**a**) and in men (**b**). In each part of the figure, red dot-points represent carriers of the *TT* genotype and blue dot-points represent *G*-allele carriers. Red lines represent regression lines for carriers of the *TT* genotype while blue lines represent regression lines for *G*-allele carriers. No significant effect of *FOXO3* genotype on IL10 levels as a function of age was observed in either men or women (*P* >0.1).

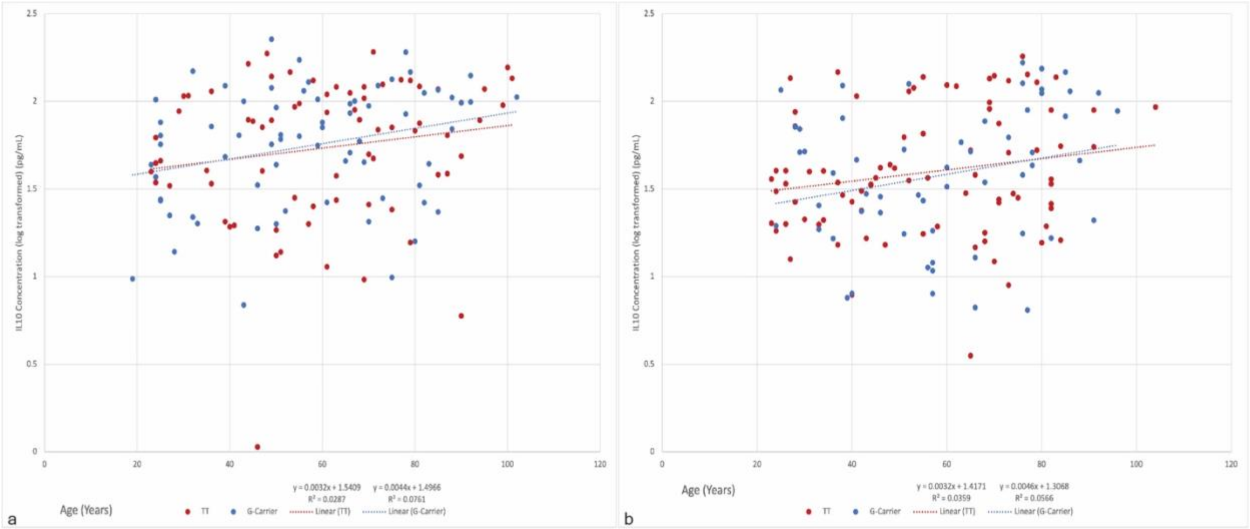

Supplementary Figure 5: Effect of *FOXO3* Genotype on Levels of IL10
IL10 cytokine levels were analyzed as function of age for each *FOXO3* genotype in women (**a**) and in men (**b**). In each part of the figure, red dot-points represent carriers of the *TT* genotype and blue dot-points represent *G*-allele carriers. Red lines represent regression lines for carriers of the *TT* genotype while blue lines represent regression lines for *G*-allele carriers. No significant effect of *FOXO3* genotype on IL10 levels as a function of age was observed in either men or women (P >0.1).
