# Supplementary material for: Novel protective effect of the FOXO3 longevity genotype on mechanisms of cellular aging in Okinawans
Source: NPJ Aging. 2024 Mar 8;10(1):18. doi: 10.1038/s41514-024-00142-8 (PMC10923797; doi:10.1038/s41514-024-00142-8)
Supplement: Supplementary file 2 — Reporting Summary [file 41514_2024_142_MOESM2_ESM.pdf]

nature portfolio

Corresponding author(s): *Double-anonymous peer review submissions: write DAPR and your manuscript number here instead of author names.*

Last updated by author(s): *YYYY-MM-DD*

Reporting Summary

Nature Portfolio wishes to improve the reproducibility of the work that we publish. This form provides structure for consistency and transparency in reporting. For further information on Nature Portfolio policies, see our Editorial Policies and the Editorial Policy Checklist.

Statistics

For all statistical analyses, confirm that the following items are present in the figure legend, table legend, main text, or Methods section.

n/a	Confirmed	
☐	☒	The exact sample size (*n*) for each experimental group/condition, given as a discrete number and unit of measurement
☐	☒	A statement on whether measurements were taken from distinct samples or whether the same sample was measured repeatedly
☐	☒	The statistical test(s) used AND whether they are one- or two-sided<br>*Only common tests should be described solely by name; describe more complex techniques in the Methods section.*
☒	☐	A description of all covariates tested
☐	☒	A description of any assumptions or corrections, such as tests of normality and adjustment for multiple comparisons
☐	☒	A full description of the statistical parameters including central tendency (e.g. means) or other basic estimates (e.g. regression coefficient) AND variation (e.g. standard deviation) or associated estimates of uncertainty (e.g. confidence intervals)
☐	☒	For null hypothesis testing, the test statistic (e.g. *F*, *t*, *r*) with confidence intervals, effect sizes, degrees of freedom and *P* value noted<br>*Give P values as exact values whenever suitable.*
☒	☐	For Bayesian analysis, information on the choice of priors and Markov chain Monte Carlo settings
☒	☐	For hierarchical and complex designs, identification of the appropriate level for tests and full reporting of outcomes
☒	☐	Estimates of effect sizes (e.g. Cohen's *d*, Pearson's *r*), indicating how they were calculated

Our web collection on statistics for biologists contains articles on many of the points above.

Software and code

Policy information about availability of computer code

Data collection	N/A
Data analysis	N/A

For manuscripts utilizing custom algorithms or software that are central to the research but not yet described in published literature, software must be made available to editors and reviewers. We strongly encourage code deposition in a community repository (e.g. GitHub). See the Nature Portfolio guidelines for submitting code & software for further information.

Data

Policy information about availability of data

All manuscripts must include a data availability statement. This statement should provide the following information, where applicable:

- Accession codes, unique identifiers, or web links for publicly available datasets
- A description of any restrictions on data availability
- For clinical datasets or third party data, please ensure that the statement adheres to our policy

All data collected and used for the analysis are available via Excel file in Supplemental Materials. The corresponding author may be contacted to request the raw data and/or materials generated during this study.

Research involving human participants, their data, or biological material

Policy information about studies with human participants or human data. See also policy information about sex, gender (identity/presentation), and sexual orientation and race, ethnicity and racism.

Reporting on sex and gender	Analysis was performed based upon sex of the participants (male and female) as determined through identification and documentation on medical records. Consistent use of sex was maintained throughout the study and analysis.
Reporting on race, ethnicity, or other socially relevant groupings	Reporting on race, ethnicity, and other socially relevant groupings were not extensively considered. All participants were self-identified as Okinawan through reporting and documentation on medical records.
Population characteristics	320 Okinawans, both male and female, were recruited ranging from the ages of 19-years-old to 104-years-old.
Recruitment	Patients were recruited during nationally required annual health screenings. Potential for bias was minimized as every patient coming in for the annual health screenings were offered the opportunity to participate. Subjects were excluded from participation if they were (a) aged <18 years, (b) had a recent medical complication, (c) exhibited severe dementia or an inability to comprehend the informed consent, (d) had a known genetic disease or disability, or (e) were restricted from participation by the subject's attending physician.
Ethics oversight	Study was performed after approval and under guidance of the Ethics Committees from Tomishiro Central Hospital (H25R008), Fukushima Medical University (#30167) and followed all relevant ethical regulations including the Declaration of Helsinki

Note that full information on the approval of the study protocol must also be provided in the manuscript.

Field-specific reporting

Please select the one below that is the best fit for your research. If you are not sure, read the appropriate sections before making your selection.

☒ Life sciences ☐ Behavioural & social sciences ☐ Ecological, evolutionary & environmental sciences

For a reference copy of the document with all sections, see nature.com/documents/nr-reporting-summary-flat.pdf

Life sciences study design

All studies must disclose on these points even when the disclosure is negative.

Sample size	320 participants were recruited, participated, and analyzed for the study.
Data exclusions	There was no data excluded
Replication	Experimentation was performed in triplicate but replicative trials were not performed.
Randomization	Participants were not randomized. Covariates were attempted to be controlled by exclusions from subject recruitment as well as increasing the size of the sample population.
Blinding	All researchers performing experimentation were blinded to subject identification and also blinded to subject genotype.

Reporting for specific materials, systems and methods

We require information from authors about some types of materials, experimental systems and methods used in many studies. Here, indicate whether each material, system or method listed is relevant to your study. If you are not sure if a list item applies to your research, read the appropriate section before selecting a response.

Materials & experimental systems

n/a	Involved in the study	
☐	☒	Antibodies
☒	☐	Eukaryotic cell lines
☒	☐	Palaeontology and archaeology
☒	☐	Animals and other organisms
☐	☒	Clinical data
☒	☐	Dual use research of concern
☒	☐	Plants

Methods

n/a	Involved in the study	
☒	☐	ChIP-seq
☒	☐	Flow cytometry
☒	☐	MRI-based neuroimaging

Antibodies

Antibodies used	Inflammatory cytokine analysis was performed using Antibodies to IL-1B, IL-2, IL-6, IL-10, and TNF-a from MilliporeSigma, Burlington, MA, and using the Milliplex MAP Human High Sensitivity T Cell Panel
Validation	From MilliporeSigma, "Based on the Luminex® xMAP® technology, our MILLIPLEX® map Human High Sensitivity T Cell Panel (Cat. no. HSTCMAG-28SK) is a 21-plex multiplexed assay kit for simultaneously detecting cytokines significant to Th1, Th2 and Th17 cells. The customizable panel enables the user to choose any number of analytes within the panel to meet specific research needs. In addition, the panel is available in a premixed-bead format as either<br>a 21-plex or a 13-plex kit, with the latter including only the Th1 and Th2 markers. This panel is available in our standard 96-well format. A 384-well format kit (and a premixed 21-plex) is also available (Cat. no. HSTC384- 28K). This kit has identical analytes to the 96-well version, but is not specifically tested in this application note. The 384-well kit version was developed for labs with higher throughput needs, and is used with the Luminex® FLEXMAP 3D® instrument." Further verification was performed and published: Lin L, Yang F, Wang Y, et al. Prognostic nomogram incorporating neutrophil-to-lymphocyte ratio for early mortality in decompensated liver cirrhosis. Int Immunopharmacol. 2018;56:58-64. doi:10.1016/j.intimp.2018.01.007

Clinical data

Policy information about clinical studies
All manuscripts should comply with the ICMJE guidelines for publication of clinical research and a completed CONSORT checklist must be included with all submissions.

Clinical trial registration	NA
Study protocol	NA
Data collection	NA
Outcomes	NA

Plants

Seed stocks	NA
Novel plant genotypes	NA
Authentication	NA
